# Fighting age-related orthopedic diseases: focusing on ferroptosis

**DOI:** 10.1038/s41413-023-00247-y

**Published:** 2023-03-01

**Authors:** Qin Ru, Yusheng Li, Wenqing Xie, Yilan Ding, Lin Chen, Guodong Xu, Yuxiang Wu, Fudi Wang

**Affiliations:** 1 Department of Health and Physical Education, Jianghan University, Wuhan, 430056 China; 2 Department of Orthopedics, Xiangya Hospital, Central South University, Changsha, 410008 China; 3 National Clinical Research Center for Geriatric Disorders, Xiangya Hospital, Central South University, Changsha, 410008 China; 4 The Second Affiliated Hospital, School of Public Health, State Key Laboratory of Experimental Hematology, Zhejiang University School of Medicine, Hangzhou, China; 5 The First Affiliated Hospital, Basic Medical Sciences, School of Public Health, Hengyang Medical School, University of South China, Hengyang, China

**Keywords:** Homeostasis, Metabolic bone disease

## Abstract

Ferroptosis, a unique type of cell death, is characterized by iron-dependent accumulation and lipid peroxidation. It is closely related to multiple biological processes, including iron metabolism, polyunsaturated fatty acid metabolism, and the biosynthesis of compounds with antioxidant activities, including glutathione. In the past 10 years, increasing evidence has indicated a potentially strong relationship between ferroptosis and the onset and progression of age-related orthopedic diseases, such as osteoporosis and osteoarthritis. Therefore, in-depth knowledge of the regulatory mechanisms of ferroptosis in age-related orthopedic diseases may help improve disease treatment and prevention. This review provides an overview of recent research on ferroptosis and its influences on bone and cartilage homeostasis. It begins with a brief overview of systemic iron metabolism and ferroptosis, particularly the potential mechanisms of ferroptosis. It presents a discussion on the role of ferroptosis in age-related orthopedic diseases, including promotion of bone loss and cartilage degradation and the inhibition of osteogenesis. Finally, it focuses on the future of targeting ferroptosis to treat age-related orthopedic diseases with the intention of inspiring further clinical research and the development of therapeutic strategies.

## Introduction

Ferroptosis, characterized a decade ago, is acknowledged as a relatively newly discovered mode of cell death.^1^ In contrast to traditional programmed cell death modalities, such as apoptosis, ferroptosis has been attributed to the biochemical characteristics of iron accumulation and lipid peroxidation. Morphologically, shrunken mitochondria are unique characteristics of ferroptosis in conjunction with densely condensed mitochondria and decreased or vanishing mitochondrial cristae.^2^ As a unique mode of cell death, ferroptosis has attracted the interest of researchers in many research fields, including those studying heart diseases,^3–6^ liver diseases,^7,8^ kidney disease,^9^ and cancers,^10^ in recent years.

Aging is a natural and irreversible process that is related to the accumulation of multifactorial degenerative processes characterized by morphological changes, functional decline, and metabolic disorders.^11,12^ With the accelerating increase in the number of elderly people worldwide, aging is becoming the most important decisive risk factor for degenerative orthopedic diseases, such as osteoarthritis, osteoporosis, and lumbar disc herniation.^13,14^ Among patients aged ≥65 years of age, degenerative orthopedic diseases are the most common causes of weakness and reduced mobility.^15,16^ When not managed appropriately, degenerative orthopedic diseases may lead to unsatisfactory outcomes, including dysfunction or mortality.^17,18^ Therefore, it is of great clinical significance to gain insight into the underlying mechanisms of degenerative orthopedic diseases and develop targeted intervention strategies. Notably, the accumulation of iron over time in organs, including cartilage, leads to increased oxidative stress and decreased normal function; moreover, ferroptosis has been linked to the onset and development of many age-related orthopedic diseases. Therefore, ferroptosis has garnered great interest in this research community, and focusing on ferroptosis may offer new therapeutic approaches for treating age-related orthopedic diseases.

Although ferroptosis research in many fields is still in its infancy, the number of published papers relating to ferroptosis in aging and degenerative orthopedic diseases has increased exponentially. Therefore, a comprehensive review and in-depth analysis of recent research progress, such as those regarding the molecular mechanisms, potential physiological functions, and possible therapeutic prospects of ferroptosis in the pathogenesis of senile degenerative orthopedic diseases, are required to clarify the principal advances and challenges to studying ferroptosis. This review offers a comprehensive description of the processes involved in systemic and cellular iron metabolism and recent advances in understanding the roles played by ferroptosis in various forms of age-related degenerative orthopedic diseases. Additionally, this review describes research on the feasibility of targeting ferroptosis to the diagnosis and treatment of these diseases and explores emerging questions and challenges in the field.

## Overview of ferroptosis

Different types of programmed cell death, including apoptosis, autophagy, and pyroptosis, have been implicated in the pathophysiology of degenerative orthopedic diseases.^19–21^ However, an increasing body of evidence reported in the past decade indicates that ferroptosis, which is characterized by the lethal accumulation of labile iron and lipid hydroperoxides, also contributes to the pathophysiology of degenerative orthopedic diseases. Ferroptosis involves the antagonism between intracellular oxidative stress and antioxidant defense systems and is induced when iron accumulation-induced oxidative stress damages cells because it significantly exceeds the antioxidant buffering capacity conferred by cellular defense systems.^22–24^

Ferroptosis can be distinguished from other traditional types of programmed cell death by the morphological and biochemical changes attributed to it.^1,25–29^ The morphological shift in mitochondria caused by reactive oxygen species (ROS) and iron overload is the most noticeable difference. Shrunken mitochondria are distinguished by densely packed condensed mitochondria and decreased or absent mitochondrial cristae.^3,7,30,31^ Ferroptosis does not cause cell membrane rupture or cell swelling, making it completely different than typical necrosis. Moreover, in contrast to apoptosis, ferroptosis does not lead to cell shrinkage, pyknosis, karyorrhexis, or the separation of cell fragments into apoptotic bodies.^32^ Biochemically, ferroptosis is characterized mainly by lipid peroxidation and is usually accompanied by excessive iron levels, increasing free radical levels, and diminished antioxidant enzyme activity.^30^ Multiple genes control the biological process of ferroptosis. For instance, genes that encode critical antioxidant enzymes, including glutathione (GSH)/glutathione peroxidase 4 (GPX4), coenzyme Q10 (CoQ10)/ferroptosis suppressor protein 1 (FSP1),^22^ nuclear factor erythroid 2-like 2 (Nrf2), dihydroorotate dehydrogenase (DHODH)^33,34^ and genes involved in lipid metabolism, such as acyl-CoA synthetase long-chain family member 4 (ACSL4), and genes encoding proteins in related pathways are considered to be specific suppressors or drivers of ferroptosis.^23,24,35–39^ Descriptions of the roles played by these biological pathways in mediating ferroptosis and age-related orthopedic diseases are presented in great detail in the following sections.

Although ferroptosis was discovered as a form of cell death 10 years ago, ferroptosis-like cell death had been described for a very long time; for example, “oxytosis” was coined described cell death caused by oxidative stress.^40,41^ From the 1950s and later, Harry Eagle’s pioneering work showed that cystine deficiency caused the death of cultured cells, and endogenous cysteine synthesis induced by glucose and methionine, known as trans-sulfation, increased cell resistance to this form of cell death.^42,43^ Prior to the introduction of ferroptosis as a form of cell death, three major areas of research converged to provide a fundamental understanding of the phenomenon was now call “ferroptosis”, namely, metabolic mechanisms, the regulation of free radicals, and iron metabolism.^44^ Among these contributors, the availability of iron is the crucial driving factor in ferroptosis, and numerous proteins involved in the maintenance of systemic and cellular iron homeostasis influence cell susceptibility to ferroptosis.

## Iron metabolism in bone and cartilage

### Systemic and cellular iron metabolism in bone and cartilage

**Fig. 1:**
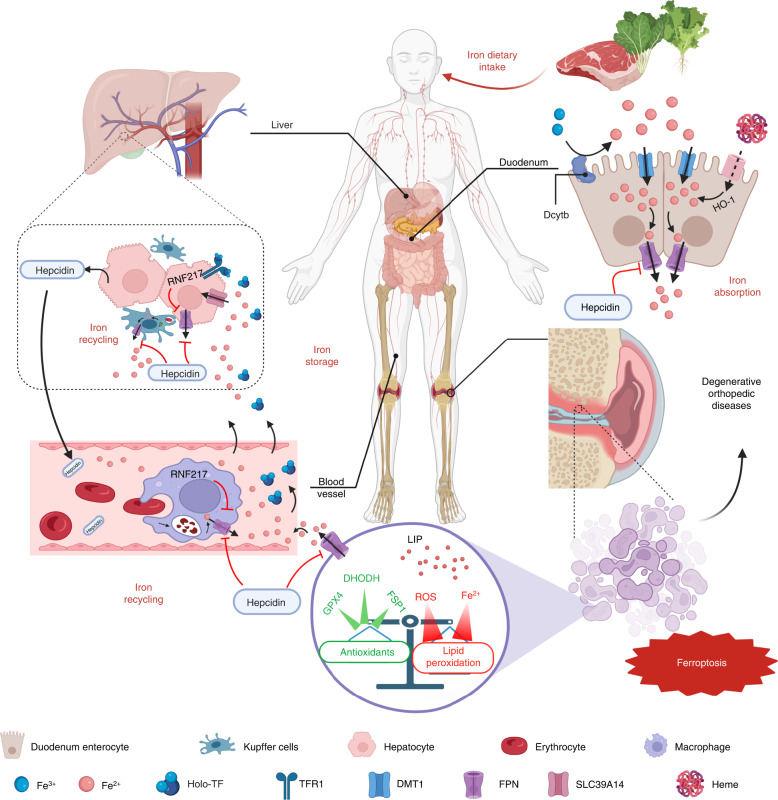
Regulation of systemic iron homeostasis. Following dietary iron intake, Fe^3+^ is reduced by duodenal cytochrome b (DcytB) and then transferred to duodenum enterocytes by DMT1. The duodenal epithelium absorbs nearly all dietary iron. Heme in the diet is degraded by HO-1 in intestinal epithelial cells after absorption. Once exported to the blood circulation by ferroportin (FPN), Fe^2+^ can be oxidized to Fe^3+^ by hephaestin, and Fe^3+^ binds to transferrin to form holo-transferrin (holo-TF, also known as TF-Fe_2_) and travels to tissues where it is abundantly utilized in target cells and is used for erythropoiesis. Macrophages in circulating blood and Kupffer cells in the liver degrade senescent erythrocytes to recycle iron. Excessive iron is stored in hepatocytes by transporters on the cell surface, such as TFR1 (Fe^3+^) or SLC39A14 (Fe^2+^), and the liver is the primary organ for iron storage. The release of intracellular iron is precisely regulated by FPN, which is the only iron exporter in the body, to maintain relatively stable iron levels. Hepcidin, a circulating hormone synthesized in the liver, is a major regulator of systemic iron homeostasis and regulates iron homeostasis by inhibiting FPN. In aging populations, iron accumulation induced by abnormal iron metabolism and excessive free radicals such as ROS results in increased lipid peroxidation in osteoblasts or chondrocytes. Similarly, a decrease in the levels of factors with antioxidant capacity, such as GPX4 and FSP1, lead to an imbalance in the intracellular oxidative stress response and antioxidant system, resulting in cell ferroptosis and ultimately the onset and progression of degenerative orthopedic diseases. Created with BioRender.com

Iron is the most abundant essential metal in mammals and plays a critical role in many physiological processes. For example, iron is involved the formation of the iron porphyrin complex known as heme, and other roles played by iron depend largely on its highly efficient electron transfer properties, which enable it to donate or accept electrons and thus act as a catalytic cofactor in various biochemical reactions.^45^ The small intestine plays a major role in dietary iron absorption by the body; specifically, the duodenal epithelium absorbs nearly all the iron that is obtained through diet. Fe^3+^ in the diet is reduced to Fe^2+^, which is more stable in the acidic environment of the stomach and duodenum, and transported into the duodenal epithelium by the divalent metal ion transporter (DMT1)^46^ (Fig. 1). Iron absorbed into the intestinal epithelium eventually enters the circulatory blood system through the conserved vertebrate iron exporter ferroportin (FPN).^47,48^ This process requires the participation of hephaestin, which oxidizes Fe^2+^ to Fe^3+^ and facilitates iron export from intestinal enterocytes into systemic iron pools.^49,50^ Under normal homeostasis, iron in systemic iron pools binds mainly to transferrin (TF) and is consumed in erythropoiesis. Senescent erythrocytes are devoured by macrophages, and the iron contained in the erythrocytes is then released into the systemic iron pool via FPN in a process called iron recycling. When the body’s iron demand increases (for example, during hematopoiesis or pregnancy), iron absorption increases; in contrast, iron overload leads to reduced iron absorption.^51^ Hepcidin, a hormone secreted by the liver, regulates FPN expression and systemic iron availability in the body.^52,53^ RNF217, an E3 ubiquitin-protein ligase, mediates the ubiquitination of FPN, thereby facilitating the degradation of FPN in the gut and spleen and regulating the absorption and recycling of iron.^54^

**Fig. 2:**
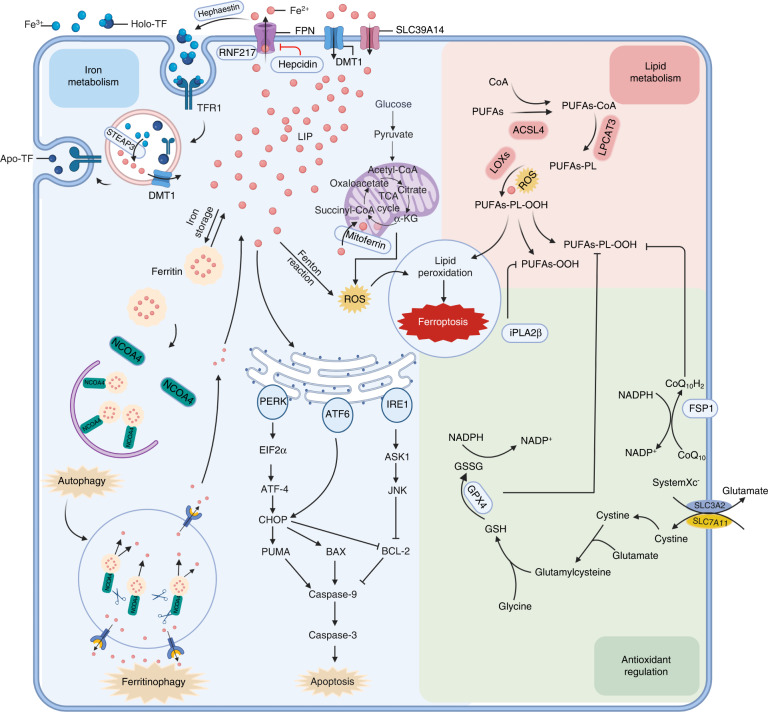
Metabolic pathways involved in ferroptosis and crosstalk between ferroptotic components and components involved in other cell death modalities. This figure mainly depicts the core network of ferroptosis regulation, which can be approximately divided into three pathways. The first pathway involves iron metabolism, including iron import and export, storage or overload. Intracellular iron can be stored in two forms: stable iron bound to ferritin or active unbound iron known as labile iron pool (LIP). Iron is usually stored in the form of cytosolic ferritin because of oxidative stress-related toxicity caused by excessive LIPs. Through Fenton chain reactions, excessive iron may rapidly amplify phospholipid hydroperoxide production, which can eventually trigger ferroptosis. The second pathway revolves around lipid metabolism involving lysophospholipid acyltransferase 3 (LPCAT3), long-chain fatty acid CoA ligase 4 (ACSL4), and other enzymes. The activation of ACSL4, LPCAT3, or lipoxygenase (LOX) in the lipid metabolism pathway accelerates lipid peroxidation and ferroptosis. The third pathway involves antioxidation regulation, including the System Xc^−^/GSH/GPX4 pathway and CoQ10/ferroptosis inhibitor protein 1 (FSP1) pathway. System Xc^−^, composed of the transporter protein SLC7A11 and regulator protein SLC3A2, exports intracellular glutamate in exchange for extracellular cystine. Using GSH as a cofactor, GPX4 reduces phospholipid hydroperoxides to corresponding alcohols. The subsequent disruption in GSH/GPX4 metabolism may induce ferroptosis. Phospholipid peroxidation is controlled by the CoQ_10_/FSP1 system or iPLA2β. These mechanisms exhibit cooperative functions leading to ferroptosis. Although ferroptosis is different from other cell death types, crosstalk between ferroptosis and apoptosis or autophagy compounds is evident. The excessive iron-activated ER stress response mediated via the activation of the PERK-EIF2α-ATF4-CHOP pathway results in increased expression of PUMA, which participates in synergistic apoptosis and ferroptosis effects. Nuclear receptor coactivator 4 (NCOA4) is a selective-cargo transporter that delivers ferritin in ferritinophagy, and the inhibition of NCOA4 inhibits ferritin autophagy-related degradation and ferroptosis. Created with BioRender.com

Cellular iron transport in bone and cartilage can be classified into three processes: intake, utilization, and efflux (Fig. 2). Mammalian cellular iron uptake is mediated via four general mechanisms: internalization of TF, nontransferrin-bound iron (NTBI), ferritin, and heme. Fe^3+^ in the form of circulating iron can be imported by cells in several ways, the most important of which involves TF, which is enriched in plasma and is a natural chelating agent with two high-affinity ferric iron-binding sites.^55^ TF-bound iron is usually internalized after docking with the transferring receptor (TFR1) on the cell membrane. TFR1 shows nanomolar affinity for iron-binding TF but no affinity for apotransferrin (apoTF; i.e., iron-free transferrin) at physiological pH.^56^ After internalization via endocytosis, Fe^3+^ is released from the TF-TFR1 complex when the pH of endosomes is reduced to 5.5, while TFR1 and apoTF are recycled to the cell membrane. The ferrireductase activity of six-transmembrane epithelial antigen of prostate 3 (STEAP3) reduces ferric iron to ferrous iron; then, ferrous iron is released into the cytoplasm by DMT1.^57,58^ Many studies have reported that the level of TFR1 is positively linked to osteoclast differentiation and function.^59–61^ The increased iron requirement of osteoclasts undergoing differentiation increases the expression of TFR1 through posttranscriptional regulation; therefore, iron intake mediated by TFR1 promotes the differentiation of osteoclasts and bone resorption.^62^ The second way cells obtain iron is through the uptake of NTBI, which is considered a “free iron” mechanism. Membrane transporters, such as DMT1, SLC39A14 (also known as ZIP14), and SLC39A8 (also known as ZIP8), and calcium channels are involved in NTBI uptake.^63–66^ Absorption of the porphyrin-bound iron of hemoglobin and heme, as well as ferritin uptake, are important mechanisms for iron internalization.^67,68^ Iron can be consumed or stored once inside cells. Mitoferrin 1 and 2 transfer iron to mitochondria, where it facilitates electron transport in the mitochondrial respiratory chain and heme synthesis.^69^ Intracellular iron is stored in two forms: stable ferritin-bound iron or active unbound iron known as the labile iron pool (LIP). Iron is typically stored in the form of cytosolic ferritin because of oxidative stress-related toxicity caused by excessive labile iron, with the liver the primary organ for iron storage. The efflux of iron from cells is almost entirely regulated by FPN, which maintains the intracellular iron balance and prevents iron-induced toxicity.^70,71^ In bones, FPN inhibits osteoclast differentiation by regulating the TFR1, NF-κB, and JNK pathways. The mRNA expression of FPN is downregulated during the initial stage of osteoclast differentiation, and decreased expression of FPN promotes osteoclast differentiation.^72^ In FPN-mutant mice, a reduction in axial bone mass was caused by a reduction in the osteogenesis rate induced by increased plasma iron in vivo,^73,74^ suggesting that FPN is a negative regulator of osteoclasts.

Iron metabolism plays crucial roles in bone remodeling, which is a complicated biological process.^75^ Osteoclasts absorb bone tissue, whereas osteoblasts produce new bone tissue. To maintain bone integrity, this process must be tightly regulated. Increasing evidence indicates that both iron overload and deficiency affect osteoclast and osteoblast differentiation and activity in a manner that disrupts the delicate balance between bone production and resorption and accelerates bone loss, suggesting that optimal iron levels are required to maintain skeletal homeostasis.^76^

Osteoclasts originate from mononuclear/macrophage myeloid lineage cells.^77^ The receptor activator of the nuclear factor κB ligand (RANKL)/osteoprotegerin (OPG) pathway regulates the differentiation and activation of osteoclasts, and the RANKL/OPG ratio is a major factor in bone integrity and bone mass.^78,79^ Osteoclasts require many mitochondria to provide energy, and mitochondrial biogenesis is an iron-dependent process. During osteoclast differentiation, key genes in heme-dependent and TF-dependent iron intake pathways are highly upregulated, and the expression of SLC39A14 (an NTBI iron transporter) and Scara-5 and Tim-2 (ferritin transporters) are either downregulated or undetectable in osteoclast lineage cells.^80^ Therefore, osteoclasts primarily obtain iron via TFR1-mediated iron uptake; furthermore, osteoclast differentiation has been demonstrated to be tightly linked to TFR1 activation and enhanced iron uptake.^61,62^ The RANKL/OPG ratio increases when iron levels are excessive, and osteoclast differentiation and bone resorption are promoted by TFR1-mediated iron absorption. In contrast, iron-chelated lactoferrin increases bone mineral density by inhibiting osteoclast bone resorption mediated by a decrease in the RANKL/OPG ratio.^81,82^ Loss of TFR1 in mature osteoclasts results in a significant reduction in total intracellular iron content (approximately 50%), whereas iron levels in monocytes and osteoclast precursor cells are affected to a lesser degree by TFR1 deficiency.^80^ Mechanistically, by activating the Src-Rac1-WAVE regulatory complex axis and inhibiting mitochondrial respiration, TFR1 depletion attenuates the cytoskeletal organization and mitochondrial metabolism of mature osteoclasts in vitro and reduced bone resorption.^80^ Several studies have shown that excessive iron-induced bone loss is due to an increased number and heightened activity of mature osteoclasts^83,84^ Together, these findings suggest that excessive iron might promote osteoclast development and activity, and both can contribute to bone resorption.

Osteoblasts differentiate from bone marrow-derived mesenchymal stem cells (BM-MSCs).^85^ The most important osteogenic transcription factor is runt-related transcription factor 2 (Runx2).^86^ Runx2-knockout mice exhibited poor bone formation and died soon after birth due to a lack of osteoblasts.^87^ Information on the direct influences of iron on osteoblasts is currently limited. Although neither Fe^3+^ nor Fe^2+^ significantly alter the levels of the majority of osteoblast differentiation markers, both Fe^3+^ and Fe^2+^ have been reported to significantly inhibit alkaline phosphatase (ALP) expression and osteoblast mineralization; furthermore, Fe^3+^ leads to more profound negative effects on osteoblast mineralization and ALP expression than those induced by Fe^2+^.^88^ Given that ALP is crucial for osteoblast mineralization, a reduction in ALP activity may contribute to the detrimental effects of Fe^3+^ and Fe^2+^ on the mineralization of osteoblasts. In addition, iron inhibits the expression of osteogenic differentiation markers in C2C12 myoblasts and BM-MSCs,^89,90^ suggesting that iron exhibited greater inhibitory effect on the expression of osteoblast differentiation markers in osteogenic precursor cells than in committed osteoblasts during osteogenic induction. Iron overload impairs BM-MSC osteogenic development and the function of mature osteoblasts. Superparamagnetic iron oxide nanoparticles (ferucarbotran) inhibited BM-MSC osteogenic differentiation by increasing the intracellular iron content, and the iron chelator deferoxamine (DFO) eliminated the anti-osteogenic effect of ferucarbotran,^91,92^ demonstrating the key function of iron in inhibiting osteogenic differentiation of BM-MSCs. Iron also inhibited the expression of Runx2 and its targets ALP and osteocalcin at a concentration of 50 μmol·L^−1^ and entirely blocked the osteogenic differentiation of BM-MSCs.^93^ Moreover, iron overload led to the downregulation of osteoblast phenotype genetic markers and decreased the formation of mineralized bone nodules.^93^ According to these findings, excessive iron exerts a negative effect on osteogenic differentiation, mature osteoblast activity and extracellular matrix mineralization. The bone loss in patients suffering from systemic iron overload is a result of decreased osteoblast bone formation and accelerated osteoclast bone resorption.

In addition to its fundamental role in oxygen delivery, iron is a cofactor for several different enzymes in the body. In terms of bone physiology, iron has been significantly associated with the formation of collagen and vitamin D metabolism, and iron deficiency has been hypothesized to negatively affect bone homeostasis.^94,95^ Studies on animal have reported a connection between bone health and dietary iron limitation and revealed that severe depletion of nutrition-derived iron significantly impacts bone health, as manifested in reduced bone mineral content, bone mineral density (BMD), and femoral strength.^96,97^ The rate of bone formation is lower than that of bone absorption; therefore, long-term iron deficiency might cause bone loss and increase the risk of osteoporosis. Clinical trials in patients with iron-deficiency-related anemia have demonstrated that mild iron deficiency enhances bone resorption.^98^ Although the use of iron-intensive nutrition interventions for women with iron-deficiency increased their iron levels, their bone formation and resorption indicators remained unchanged.^99^ In contrast, Zhao et al. reported that low iron concentrations exerted biphasic effects on osteoblasts, with mildly low iron levels promoting osteoblast activity and very low iron levels inhibiting osteoblast activity; osteogenic effect was the best when iron levels reached specific concentration.^100^

The third type of cell in bones, the osteocyte, is found within the bone matrix and is the most abundant cell in bone tissue. Osteocytes are considered to be the main coordinators of bone activity, as they can sense and integrate chemical and mechanical signals in their microenvironment to control bone formation and bone resorption; notably, studies have increasingly recognized a significant role for osteocytes in bone remodeling and bone homeostasis.^101^ Purified osteocytes expressed a higher level of RANKL than osteoblasts and BM-MSCs, and they exhibited a greater ability to support osteoclast genesis in vitro, according to Nakashima et al.^102^. However, information to determine whether excess or deficient iron affects the activities and function of osteocytes is lacking, and previous studies on bone homeostasis have focused mainly on osteoclasts and osteoblasts. In primary cultured osteocytes and MLO-Y4 cells (osteocyte-like cells), iron overload induced an increased apoptosis rate, which promoted osteoclast differentiation by promoting RANKL secretion.^103^ Recent evidence has shown that osteocytes and their lacunae exhibit morphological changes in aging bones, indicating that osteocytes are involved in the process underlying degenerative bone aging.^101^ Greater understanding of osteocyte mechanisms may help to identify promising targets and effective therapies for the treatment of age-related orthopedic diseases.

The evidence suggests that the delicate balance between bone production and resorption is disrupted by both iron overload and iron deficiency and that optimal iron levels are needed to maintain bone homeostasis; however, it is unclear which change (iron excess or iron deficiency) may exert a more serious effect on bone health. Further research is required to better understand how excessive or deficient iron levels affect the differentiation and function of osteoclasts, osteoblasts, and osteocytes.

### Regulation of systemic and cellular iron metabolism in the bone and cartilage system

To ensure that the body has enough iron for the maintenance of physiological activities and to prevent toxic iron accumulation, systemic iron homeostasis is controlled in a sophisticated manner. Hepcidin is the main factor in the maintenance of iron homeostasis.^104^ Hepcidin induces the internalization and degradation of FPN,^52,53,105^ which inhibits iron export to the blood circulatory system, particularly iron in duodenal enterocytes, hepatocytes, and macrophages. During iron overload, the liver synthesizes and secretes more hepcidin, which accelerates FPN degradation and thus leads to a decrease in the amount of iron exported into the circulatory system. Hepcidin expression is decreased during increased iron demand or absolute iron deficiency, and the suppression of hepcidin expression promotes iron absorption and recycling to maintain the body’s iron homeostasis.^52,105^

Furthermore, hepcidin levels are regulated by hypoxia and inflammatory factors. For example, hypoxia-inducible factor 1 (HIF1) and HIF2 in the liver inhibit hepcidin expression,^106,107^ and HIF activation is regulated by intracellular iron via the modulation of hydroxylase activity, which requires iron as a cofactor.^108^ Reduced hepcidin expression can increase iron absorption and enhance hematopoiesis. Notably, administration of erythropoietin in mice reduced hepcidin levels in a dose-dependent manner, an outcome that has been confirmed in human clinical trials.^109^ In patients with severe anemia, the hematopoietic system was activated through feedback to upregulate the erythrocyte regulatory factor erythroferrone (ERFE), inhibit the expression of hepcidin, and increase iron absorption. ERFE-knockout mice were unable to inhibit hepcidin production rapidly after hemorrhage and recovered slowly after blood loss.^110^ In contrast, ERFE overexpression resulted in iron overload and developmental abnormalities in mice.^111^ Hepcidin expression is also upregulated during inflammation; for instance, interleukin-6 (IL-6) directly regulates hepcidin expression by activating signal converter and activator of transcription 3,^112^ and the bone morphogenetic protein signaling pathway is required for IL-6-induced hepcidin expression.^113^

The posttranscriptional regulation of iron-regulated protein 1 (IRP1) and iron regulatory protein (IRP2) is critical for regulating cellular iron metabolism. IRP1 regulation depends on iron–sulfur clusters within mitochondria. When the intracellular iron level is high, IRP1 binds to iron–sulfur clusters, which prevents IRP1 from binding to iron-responsive elements (IREs), and thus acts as a cytosolic aconitase. Furthermore, when the cellular iron level is deficient, IRP1 loses the ability to bind with iron–sulfur clusters and to exhibit aconitase activity; in contrast, it combines binds an IREs to play a positive regulatory role in iron homeostasis; hence, IRP1 is a bifunctional regulatory factor.^114^ That is, the specific function of IRP1 depends on the utilization of mitochondrial iron and synthesis of iron–sulfur clusters, whereas IRP2 mainly senses intracellular iron levels. Compared to wild-type mice, IRP2^−/−^ mice showed obvious symptoms of osteoporosis, such as decreased bone mineral density and bone iron deficiency.^115^ The levels of bone formation markers in serum, including bone-gla-protein and type I collagen, were decreased, whereas those of bone resorption markers, such as tartrate-resistant acid phosphatase and cathepsin K, were significantly elevated.^115^ In the bone tissue of IRP2^−/−^ mice, the expression of ferritin and FPN was reduced, while TFR1 expression was elevated, suggesting that iron disorders in bones may lead to osteoporosis.

### Abnormal iron metabolism and age-related orthopedic diseases

Iron homeostasis is profoundly impacted by aging. Recent research has revealed that elderly people suffer from cellular iron accumulation, which can result in a variety of tissue degeneration and diseases. Cellular iron deposition caused by aging can occur in various types, such as ferritin, lipofuscin, and labile iron. The age-related increase in ferritin is frequently attributed to systemic inflammation, which is driven by aging. Elderly people tend to present with high levels of ferritin, even when they also present with iron deficiency due to inflammation, indicating that ferritin is a sensitive biomarker of age-related inflammation.^116^ The iron concentration in senescent cells is 10-fold higher than that in young cells, and elevated levels of cellular ferritin are thought to be powerful indicators of cellular senescence and aging.^117^ The age-related accumulation of lipofuscin is also catalyzed by labile cellular iron. As intralysosomal aggregates are composed primarily of oxidized proteins and lipids, lipofuscins, which are resistant to ubiquitin–proteasome degradation, often accumulate during aging and induce cytotoxic effects via the persistent generation of oxidants.^118^

Osteopenia and osteoarthritis are both influenced by age-related dysregulation of iron homeostasis.^75,119^ For instance, excessive systemic iron levels result in cellular iron accumulation in the knee joint, leading to higher levels of local inflammatory mediators, which are associated with the early pathogenesis and progression of knee osteoarthritis.^120^ Moreover, the proinflammatory cytokines TNF-α and IL-1β disrupt iron homeostasis in chondrocytes by promoting iron influx, which leads to chondrocyte iron overload and subsequently increases the levels of chondrocyte catabolic markers, such as matrix metalloproteinases 3 (MMP3) and MMP13, and promotes iron overload-induced cartilage degeneration, which can be inhibited by reducing iron concentrations with iron chelators or antioxidant drugs.^121,122^ Iron metabolism disorders, especially iron overload, can lead to bone loss and are associated with osteoporosis. Reducing iron levels in bone tissue inhibits osteoclast differentiation and bone resorption.^123^ Excessive iron promoted oxidative stress, which caused trabecular bone damage and the imbalance in bone homeostasis, leading to bone loss in mice; furthermore, antioxidants such as resveratrol significantly inhibited subsequent osteoclastogenesis.^124^

Under the influence of different induction factors, BM-MSCs differentiate into osteoblasts or chondrocytes. Therefore, BM-MSCs show clear importance in bone and cartilage development and reconstruction and in tissue regeneration. Disrupted cellular iron metabolism in BM-MSCs abrogated their proliferation and differentiation balance, severely damaged the bone marrow microenvironment in mice, and promoted osteoclast differentiation and bone resorption.^125^ Intra-articular anti-inflammatory and proinflammatory cytokines maintain dynamic equilibrium and jointly maintain the physiological metabolism of articular cartilage. The accumulation of iron in synovial cells can induce a cytokine storm in joints, which causes chronic synovitis. This cascade increases the ability of intra-articular monocytes and synovial fibroblasts to respond to TF-bound iron uptake and NTBI uptake, accelerating the degradation of connective tissues mediated by cathepsins released by chondrocytes and intravascular tissues, ultimately leading to destructive joint disease.^126,127^

## Ferroptosis in age-related orthopedic diseases

### Ferroptosis exacerbates osteoporosis

**Fig. 3:**
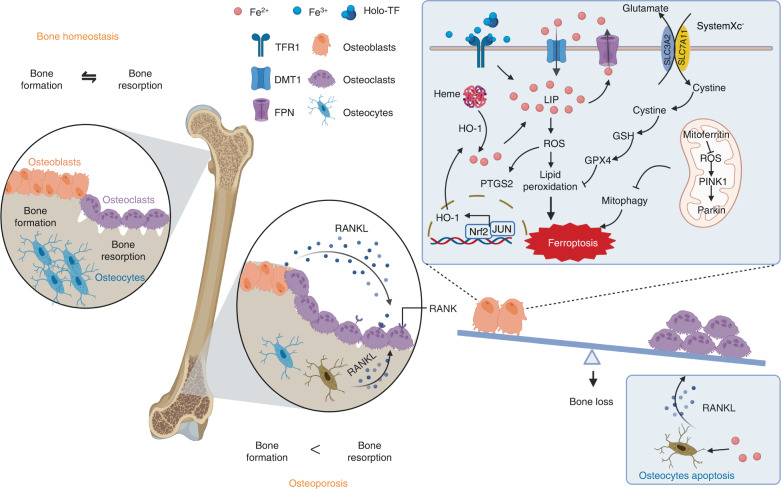
Regulatory role of ferroptosis in the onset of osteoporosis. Bones are metabolically active tissues that are constantly being remodeled by osteoclasts, which resorb mineralized bone, and osteoblasts form new bone. Iron overload can induce osteocyte apoptosis and effectively promote osteoclastogenesis by facilitating the secretion of a pro-osteoclastogenic cytokine known as receptor activator of nuclear factor κB ligand (RANKL). Iron overload exerts a significant impact on the survival of osteoblasts, resulting in a decrease in their mineralization capacity. Excessive iron in osteoblasts can increase the expression of DMT1, TFR1, and prostaglandin endoperoxide synthase 2 (PTGS2) and decrease the expression of SLC7A11 and GPX4, which accelerates lipid peroxidation and causes osteoblast ferroptosis. Moreover, the Nrf2:c-JUN heterodimer promotes the transcription of heme oxygenase-1 (HO-1). HO-1 catalyzes the oxidation of heme to release a large amount of labile iron and catalyzes the Fenton reaction to form lipid peroxides, which accelerates the ferroptosis of osteoblasts. In addition, mitochondrial ferritin plays a role in osteoblast ferroptosis; moreover, mitochondrial ferritin (mitoferritin) stores iron in mitochondria. Silencing mitoferritin induces mitophagy via the ROS/PINK1/Parkin pathway, and overexpression of mitoferritin inhibits ferroptosis in osteoblasts. The ferroptosis of osteoblasts causes an imbalance in bone metabolism, ultimately leading to osteoporosis. Created with BioRender.com

Bones are metabolically active tissues that are constantly remodeled by osteoclasts, which resorb mineralized bone, and new bone is formed by osteoblasts. During growth and adolescence, bone formation is favored; however, bone density begins to decline in people 30 years old and older. Previous studies have suggested that osteoporosis is primarily caused by abnormal calcium metabolism. Nevertheless, an increasing number of clinical studies, particularly studies on menopausal women, have convincingly shown that excessive iron is associated with osteoporosis. Iron overload has been linked to bone loss and closely associated with osteoporosis.^128^ Under iron-overload conditions, both decreased bone formation and increased bone resorption are associated with pathological bone loss (Fig. 3).

Hereditary illnesses collectively known as hemochromatosis are characterized by an increase in dietary iron consumption, which may result in severe iron overload in tissues in certain patients. To date, hereditary hemochromatosis has been classified into five kinds diseases that are caused by mutations in different genes involved in iron metabolism.^129,130^ Numerous clinical studies have demonstrated the link between hemochromatosis and osteoporosis, and approximately 25%–34% of patients with hemochromatosis present osteoporosis, with 40%–80% of these patients presenting with osteopenia; moreover, the development of osteoporosis in patients with hemochromatosis has been shown to be related to the degree of iron overload.^131^ Iron overload can increase the toxicity induced by iron in joints and bones, even when the iron levels are manageable; furthermore, the regulation of intracellular iron levels and ferroptosis might be the main driving factors of bone-mass reduction in patients with hemochromatosis.^132^ Patients with thalassemia are regularly treated with blood transfusions to maintain adequate hemoglobin levels; however, repeated transfusions can lead to iron overload because the body lacks an active mechanism to expel the excessive iron it is receiving. Despite the use of iron chelation therapy, Mediterranean anemia is related to osteoporosis, and fractures are one of the most common complications in patients with this type anemia.^133^ A multiple clinical cohort study showed an increased incidence of nontraumatic fractures in patients with thalassemia, and the risk of fractures was associated with the severity of the anemia and frequency of transfusion.^134,135^ The results of a 19-year retrospective longitudinal study showed that thalassemia patients with iron overload exhibited a significant and sustained decrease in femoral neck and overall bone density, and male patients showed a greater decline in BMD and a higher risk of fracture than female patients.^136^ Further mechanistic studies have revealed that increased bone resorption due to ferroptosis is linked to the pathogenesis of thalassemia-associated bone diseases.^132,137^

Iron overload exerts a significant impact on the bone mineralization process; moreover, the death of osteoblasts is significantly increased by iron overload, resulting in a decrease in mineralization capacity. When the iron load is excessive, iron accumulates primarily in the bone marrow, along with the liver and muscles.^138^ Iron accumulates first in osteoids, influencing the terminal differentiation of osteoblasts, and osteocalcin is significantly downregulated.^138,139^ Iron exposure can increase intracellular iron levels in osteoblasts and osteocytes, leading to cell ferroptosis.^138^ When deposited iron reaches toxic levels, the expression of DMT1 and TFR1 in osteoblasts and osteocytes increases significantly, accompanied by enhanced osteoclast activity and diminished osteoblast activity.^60,140^ Furthermore, iron overload impairs the proliferation and differentiation balance of BM-MSCs, which negatively affects bone homeostasis. Iron and exogenous ferritin specifically inhibit the expression of the osteogenic transcription factor Runx2 and its targets, including osteocalcin and ALP, to inhibit the osteogenic commitment and differentiation of BM-MSCs.^90^

A strong link between ferroptosis and glucolipid metabolism was identified in an animal model of type 2 diabetes-induced osteoporosis and may be the key mechanism of osteoblast cell death in mice with diabetic osteoporotic (DOP). Serum ferritin levels were significantly increased in the DOP mice, and the expression of SLC7A11 and GPX4 was markedly attenuated in the bone tissue of diabetic rats with bone loss. High glucose and palmitic acid levels not only inhibit osteoblast differentiation and mineralization, but also trigger osteoblast death associated with ferroptosis.^141^ DOP mice showed significant trabecular degeneration and osteopenia, which was accompanied by decreased expression of GPX4 and increased expression of prostaglandin endoperoxide synthase 2 (PTGS2), an enzyme that accelerates lipid peroxidation, implying that ferroptosis may be a mode of osteocyte death.^141^ Other studies have demonstrated that ferroptosis induced by high glucose in osteocytes is mediated by HO-1, which catalyzes the oxidation of heme to release a large amount of labile free iron and then catalyzes the Fenton reaction to form lipid peroxides. Furthermore, this process depends on the direct combination of the Nrf2:c-JUN heterodimer, and targeting HO-1 or ferroptosis can successfully reverse osteocyte ferroptosis in the diabetic microenvironment by breaking the vicious cycle of HO-1 activation and lipid peroxidation, ultimately attenuating osteoporosis.^128,142^

Mitochondrial ferritin (mitoferritin) is critical for trapping and storing toxic iron in mitochondria. Under high-glucose conditions, overexpression of mitoferritin inhibits ferroptosis in osteoblasts by reducing oxidative stress caused by excessive iron, whereas silencing mitoferritin induces mitophagy through the ROS/PINK1/Parkin pathway.^143^ These findings suggest that ferroptosis in osteoblasts might be the primary cause of type 2 diabetes-induced osteoporosis and that mitoferritin may be a potential target for clinical therapy. Alterations in the expression of ferroptosis biomarkers, such as GPX4, SLC7A11, and SLC3A2, have been observed in the steroid-induced osteoporosis context. Furthermore, extracellular vesicles derived from bone marrow-derived endothelial progenitor cells (BM-EPCs) alleviated pathological changes in steroid-induced osteoporosis, as indicated via microtomography, with a decrease in the structure model index and attenuated trabecular separation, increasing bone volume, trabecular thickness, and trabecular connectivity and density.^144^ These data confirmed that osteoporosis may be prevented by inhibiting the ferroptosis pathway in osteoblasts, which may be helpful for the potential clinical treatment of osteoporosis.

### Ferroptosis triggers osteoarthritis

**Fig. 4:**
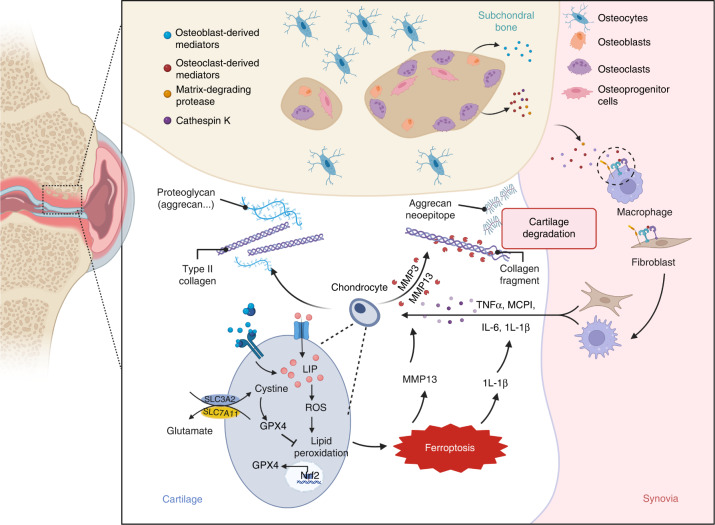
Ferroptosis triggers osteoarthritis. Cartilage loss in osteoarthritis is primarily due to an imbalance between the anabolic activity of chondrocytes (including the secretion of extracellular matrix elements, such as type II collagen and proteoglycans, which is reduced in osteoarthritis) and catabolic activity (activity of extracellular matrix-catabolic enzymes, such as matrix metalloproteinases and collagenases, which is upregulated in osteoarthritis). Subchondral bone remodeling is another key characteristic of osteoarthritis. The release of various mediators and the increase in bone resorption are driven by the activation of osteoclasts in subchondral bone, while some pro-catabolic mediators are also released by osteocytes or osteoblasts. Macrophages and fibroblasts in the synovium can secrete mediators (e.g., proinflammatory cytokines) that diffuse into articular cartilage and lead to cartilage damage. Iron overload in chondrocytes causes increased intracellular ROS levels and downregulation of GPX4, which further increases the sensitivity of chondrocytes to oxidative stress; promotes the release of inflammatory factors, such as IL-1β, and matrix metalloproteinases, such as MMP13; aggravates extracellular matrix degradation; and accelerates the progression of osteoarthritis. Created with BioRender.com

The most prevalent type of joint illness and the major cause of chronic disability among elderly people is osteoarthritis. Iron overload in joints has been observed in patients with osteoarthritis and is closely associated with multiple clinical alterations leading to osteoarthritic phenotypes, including persistent joint inflammation, degenerative cartilage degradation, synovial pannus, and proliferative synovitis.^145,146^ Clinical findings have revealed that the degree of iron overload affects the progression of osteoarthritis (ferritin concentration >1 000 μg·L^−1^), and patients with significantly elevated ferritin levels may experience severe joint complications. Imaging analysis has shown a positive correlation between ferritin levels and the severity of arthritis.^147,148^ In addition, elderly people store iron at higher levels than young people, and this contrast is more noticeable in elderly female than in male patients, which may be linked to estrogen deficiency in postmenopausal women.^149,150^ Serum ferritin concentrations were 2–3 times higher in postmenopausal women than in nonmenopausal women, and increased serum ferritin was inversely correlated with estrogen levels, which may explain the higher incidence and susceptibility of osteoarthritis in elderly females compared to males, and this difference may be mainly due to iron overload^151–153^ (Fig. 4).

Patients with hemophilia often develop severe arthritis. In hemophilic arthropathy, erythrocytes from circulating plasma enter the synovial cavity and release factors such as methemoglobin and iron. When the iron content in the synovial membrane is low, synovial cells undergo self-regulation to adapt. However, in cases of ongoing or frequent bleeding, the synovium cannot continuously to absorb or remove red blood cells, resulting in the accumulation of excessive iron in hemosiderin form, which gradually develops into synovitis.^154,155^ Iron accumulation within a joint is also observed in hemochromatosis arthropathy;^156^ furthermore, cartilage homeostasis is altered by excessive iron in patients with hereditary hemochromatosis, and the rate of type II collagen renewal is increased after excessive iron is eliminated.^157^ The Kellgren–Lawrence grade and the joint-space-narrowing score showed a U-shaped association between the progression of knee osteoarthritis and iron intake, indicating that adequate iron intake was desirable for knee osteoarthritis, whereas an excessive or deficient level of iron intake increased the risk, with an inflection point evident approximately on 16.5 mg·d^−1^.^158^ A weighted median analysis based on large-scale genome-wide association studies (GWASs) showed that serum iron, ferritin, and transferrin saturation was positively correlated with knee osteoarthritis.^159^ These observational clinical investigations have all shown an association between iron status and osteoarthritis.

Animal experiments have also revealed that iron overload accelerates osteoarthritis progression.^160^ An iron-deficient diet induced systemic iron reduction in Hartley guinea pigs, which are prone to primary osteoarthritis, that delayed the onset and/or development of cartilage lesions. The iron concentrations in the liver, serum, and articular cartilage of the Hartley guinea pigs fed an iron-deficient diet were all reduced. Increased secretion of 4-HNE, a product of lipid peroxidation mediated by iron, is hypothesized to contribute to the pathogenesis of osteoarthritis.^161^ A reduction in 4-HNE in the knee cartilage and meniscus of animals with iron deficiency was highly correlated with chondrocyte density and iron concentration, indicating that lower systemic iron concentrations may facilitate iron level regulation in the knee joint, reduce ROS production, lipid peroxidation and tissue damage.^162^ Primary chondrocytes acquire cellular phenotypes similar to those observed in osteoarthritis when exposed to excess exogenous iron, such as that associated with enhanced metalloproteinase content and reduced levels of extracellular matrix proteins, whereas articular chondrocytes isolated from homeostatic iron regulator (HFE)-knockout mice showed higher sensitivity to intracellular iron content than those from wild-type mice.^163^ These results indicate that high iron exposure may impair chondrocyte metabolism, and the loss of HFE function accelerates chondrocyte metabolic dysfunction, making it easier to acquire an osteoarthritis-related phenotype. Injection of exogenous iron into guinea pigs with a low incidence of osteoarthritis caused knee osteoarthritis in these animals. Furthermore, exogenous iron (iron dextran) altered the expression of cytokines, iron transporters, and structural components in cartilage, increasing the incidence of osteoarthritis in guinea pigs with a low risk of osteoarthritis.^120^ These findings suggest that iron overload is closely associated with the development and progression of osteoarthritis.

Further molecular and cellular studies have revealed specific mechanisms by which ferroptosis affects the onset and development of osteoarthritis. GPX4 expression in the cartilage tissue of patients with osteoarthritis was found to be significantly lower than in that of healthy people.^164^ Furthermore, GPX4 downregulation increases chondrocyte sensitivity to oxidative stress, aggravates extracellular matrix degradation, and accelerates osteoarthritis progression.^164^ Moreover, inflammation and iron overload both caused chondrocyte ferroptosis and cartilage degradation, which was accelerated by the ferroptosis inducer erastin, whereas the ferroptosis inhibitor ferrostatin-1 (Fer-1) attenuated IL-1β- and ferric ammonium citrate-induced lipid peroxidation and ferroptosis-related protein changes and promoted Nrf2 system activation.^122^ Many natural products with antioxidant or anti-inflammatory activity, including D-mannose, stigmasterol, and icariin, slow cartilage degradation by activating the GPX4 pathway or reducing lipid peroxidation to inhibit ferroptosis, thereby delaying the development of osteoarthritis.^165–167^ Therefore, activating the System Xc^−^/GPX4 axis to inhibit ferroptosis or suppress chondrocyte sensitivity to ferroptosis can be exploited as a novel therapeutic strategy for osteoarthritis.

In addition to causing cartilage degradation, ferroptosis can affect subchondral bone homeostasis and structural integrity, which are critical to the occurrence and development of osteoarthritis. Subchondral bone undergoes dynamic bone remodeling under normal circumstances, and the bone remodeling is regulated and maintained in balance by a combination of osteoclasts and osteoblasts. Additionally, TFR1-dependent iron uptake promotes osteoclastogenesis and bone resorption capacity, which are hindered by the iron chelator DFO in a dose-dependent manner.^62,168^ Excessive iron can upregulate the level of DMT1, increase intracellular iron levels and ROS production, and induce autophagy and apoptosis in osteoblasts.^169^ These results suggest that abnormal subchondral bone formation and excessive resorption of subchondral bone might be involved in the underlying mechanism of ferroptosis-related subchondral bone instability and osteoarthritis.

### Ferroptosis aggravates lumbar disc herniation

**Fig. 5:**
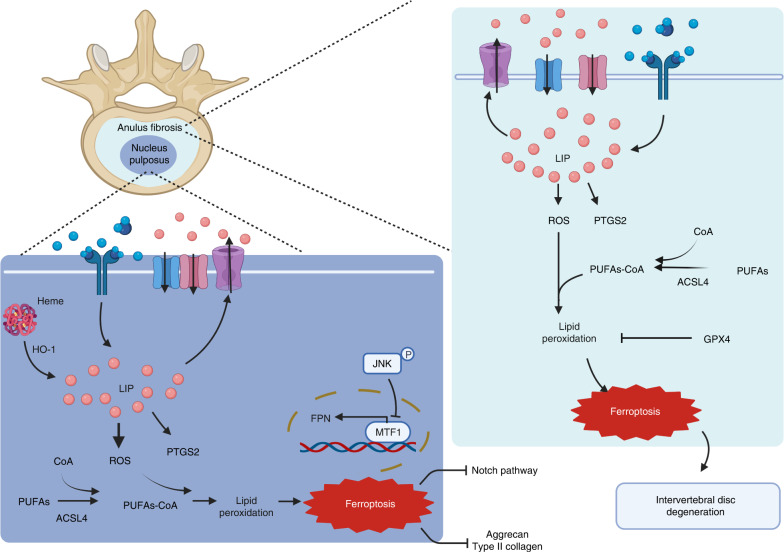
Role of ferroptosis in lumbar disc herniation. Normal intervertebral discs consist of the external annulus fibrosus (AF), which forms a ring structure surrounding the nucleus pulposus (NP). Intervertebral disc degeneration, which is affected by various factors, is the primary cause of lumbar disc herniation. Compared with healthy people, patients with intervertebral disc degeneration present with lower levels of FTH and GPX4 in the AF and NP, while their levels of PTGS2 and ROS are upregulated, which leads to lipid peroxidation accumulation in the AF and NP cells, as well as the main morphological feature of ferroptosis. In NP cells, the increased levels of cellular iron and HO-1 exacerbate lipid peroxidation, leading to cellular ferroptosis and extracellular matrix degradation and further intervertebral disc degeneration. Furthermore, increased nuclear translocation of metal-regulated transcription factor 1 (MTF1) restores FPN function, eliminates intercellular iron overload, and protects NP cells from ferroptosis. Created with BioRender.com

A prevalent cause of chronic low back pain is lumbar disc herniation, which affects 70%–85% of the population worldwide. The incidence of disc herniation is showing an increasing trend as the population ages, and 11% of patients with lumbar disc herniation experience severe disability.^170,171^ Located between vertebrae, intervertebral discs are fibrocartilaginous tissues that act as shock absorbers by promoting the of the trunk and distributing mechanical loads along the spine.^172^ Normal intervertebral discs consist of the nucleus pulposus (NP) and the external annulus fibrosus (AF), which forms a ring structure that surrounds the NP and is linked to the neighboring vertebral body via cartilaginous endplates. The AF and NP play crucial roles in maintaining intervertebral disc function.^173^ Intervertebral disc degeneration is the primary cause of lumbar disc herniation, which is mediated by various factors, such as age.^174^ The etiology of intervertebral disc degeneration includes rupture of the AF, oxidative stress in nucleus pulposus cells (NPCs) and degeneration of cartilage endplates;^175^ recent research has suggested that ferroptosis may also be implicated in this process (Fig. 5).

Compared with that in healthy people, the expression of GPX4 and ferritin heavy chain (FTH) in the disc tissue of patients with intervertebral disc degeneration was downregulated, and the expression level of PTGS2 was upregulated, suggesting that ferroptosis is involved in degenerative disc tissues.^176^ Iron overload promotes the degeneration of intervertebral discs and cartilage endplates in a dose-dependent manner.^177^ Treatment with tert-butyl hydroperoxide (TBHP), which can simulate oxidative stress conditions, results in increased levels of PTGS2 and ACSL4, accelerated lipid peroxidation and decreased expression of FTH and GPX4 in AF cells and NPCs. Additionally, the primary morphological characteristics of ferroptosis, such as small and dense mitochondria and high mitochondrial membrane density, have also been observed, and all these effects were reversed by treatment with the ferroptosis inhibitors Fer-1 and DFO.^176,177^ DFO treatment delayed the development of disc degeneration by inhibiting ferroptosis, demonstrating that ferroptosis participates in the pathogenic mechanism underlying intervertebral disc degeneration, with AF cells and NPCs the key cell types involved.

The NP is essential to the pathogenic process of disc degeneration, and NPCs constitute the main cell type critical to the synthesis of extracellular matrix component, including type II collagen and aggrecan. Single-cell RNA sequencing (scRNA-seq) results revealed that multiple genes were differentially expressed in patients with intervertebral disc degeneration compared with those healthy individuals, and GO and KEGG analyses revealed that ferroptosis pathways were enriched with these differentially expressed genes; moreover, compared with healthy individuals, patients with intervertebral disc degeneration showed reduced levels of type II collagen and aggrecan in the intervertebral disc.^178^ The results of animal experiments showed that the levels of iron and HO-1 in the NP of the model group were considerably higher than those in the control group,^178^ implying that ferroptosis may participate in the development of disc degeneration and may be a novel target for intervening in intervertebral disc degeneration. Clinical epidemiological studies have shown that serum homocysteine (Hcy) levels are markedly higher in the intervertebral disc degeneration group than in the normal group, and Hcy levels are positively correlated with clinicopathological grades, suggesting that the serum Hcy level is an independent risk factor for human intervertebral disc degeneration.^179^ These results confirm the association between intervertebral disc degeneration and NPC ferroptosis. However, little research has been performed to identify the precise mechanism of ferroptosis in the etiology of intervertebral disc degeneration. Perl staining and ferritin immunohistochemical staining of human NP tissues showed more iron deposition in the intervertebral disc degeneration group than in the control group. Treatment with the ferroptosis inhibitors liproxstatin-1 or Fer-1 and the iron chelator DFO markedly reversed the effects of TBHP-induced ferroptosis in human NPCs, which had largely been caused by cellular iron overload and lipid ROS accumulation.^180^ Further mechanistic studies have implicated FPN dysregulation as a cause of intercellular iron overload in the intervertebral disc degeneration context, and siRNA knockdown of FPN expression led to increased intercellular concentrations of iron in NPCs, whereas FPN overexpression attenuated iron overload and ferroptosis in NPCs.^180^ Furthermore, FPN was found to be essential for preserving iron homeostasis and preventing ferroptosis in human NPCs, and nuclear translocation of metal-regulated transcription factor 1 (MTF1) promoted the transcription of FPN and restored FPN function to eliminate intercellular iron overload, thereby protecting NPCs from ferroptosis.^180^

Cartilage, which regulates the diffusion of nutrients between a disc and vertebral body, is also important for maintaining the normal function of intervertebral discs. The number of chondrocytes in the endplate is greatly reduced in degenerated disc cartilage, resulting in a decrease in disc cartilage elasticity.^181^ Depending on its concentration, iron can exert either beneficial or detrimental effects on osteogenesis. Ferric ammonium citrate (FAC) dramatically increased the levels of the markers of chondrocyte degeneration, namely, COL10, RUNX2, MMP3 and MMP13, and accelerated the degeneration of endplate chondrocytes in a dose-dependent manner (10–100 μmol·L^−1^).^177^ Further mechanistic studies showed that GPX4 and SLC7A11 levels decreased in the number of endplate chondrocytes of an iron overload model in an FAC dose-dependent manner, while the expression of lipid peroxides (4-HNE) increased, accompanied by a markedly increase in the number of condensed mitochondria, suggesting that excessive iron promoted endplate chondrocyte mitochondrial dysfunction and ferroptosis. These changes were rescued by DFO, as well as the ferroptosis inhibitors Fer-1 and N-acetylcysteine.^177^ Compared to that in normal tissues, the level of miR-10a-5p was remarkedly reduced in degenerative intervertebral disc cartilage, and the expression of its target gene, IL-6R, was upregulated.^181^ Moreover, GPX4 expression was significantly lower in diseased cartilage, and IL-6 induced human chondrocyte death through ferroptosis,^181^ suggesting that an abnormal IL-6/miR-10a-5p/IL-6R axis is associated with intervertebral disc degeneration in clinical trials, and chondrocyte loss in degenerative intervertebral discs may be caused by ferroptosis.

Neovasculogenesis is another characteristic of herniated lumbar discs. The histological feature of an injured disc includes the formation of vascularized granulation tissue along pathological tears in the tissue extruding from the NP toward the AF. Histological staining revealed new blood vessels in herniated NPs of lumbar discs in patients, and this vascularization was closely related to age and disease course. Angiogenesis may be a repair process following disc injury and may contribute to tissue degeneration.^182^ Notably, the hemoglobin signal was found to be significantly increased in herniated NPs compared with nonherniated NPs, and more dark iron granules were observed in the NP of herniated discs stained with Prussian blue, consistent with results showing high tissue iron content.^183^ Immunohistochemical studies also showed that HO-1 was expressed at higher levels in the NP of herniated discs than in the NP of nonherniated discs.^183^ High levels of hemoglobin and heme led to ferroptosis in herniated tissue, and heme-induced ferroptosis was accompanied by significant changes in the mRNA and protein expression of molecules related to the Notch signaling pathway.^183^ Notably, DFO cotreatment effectively rescued heme-induced ferroptosis and inhibition of Notch pathway activation,^183^ suggesting that heme-induced cell death is iron dependent and that the neovascularization in the NP of herniated discs may promote NP degeneration through heme-induced ferroptosis, which may be related to the Notch signaling pathway.

## Targeting ferroptosis to diagnose or treat age-related orthopedic diseases

Ferroptosis can be initiated or prohibited by small compounds or drugs, as well as by changes in physiological conditions. Table 1 lists the numerous ferroptosis inducers and ferroptosis inhibitors that have been demonstrated to influence cellular iron levels, lipid peroxidation product accumulation and antioxidant enzyme activity.^184–191^ Ferroptosis can be stimulated via four different pathways. Class 1 ferroptosis inducers are characterized by the inhibition of System Xc^−^, which reduces cystine import. Blocking this system impairs cystine import, causes GSH depletion, and inactivates GPX4. Erastin, sulfasalazine, sorafenib, artesunate, glutamate, and lanperisone^192–194^ are examples of Class 1 ferroptosis inducers that target System Xc^−^.^195^ Class 2 ferroptosis inducers are characterized by their induction of GPX4 inhibition. A representative molecule, RSL3, covalently binds with GPX4 to inhibit its activity.^196^ Acetaminophen and withaferin A induce ferroptosis through a similar mechanism.^197,198^ FINO2 inactivates GPX4 indirectly by oxidizing iron and driving lipid peroxidation. The cells then accumulate lethal lipid peroxides due to dysfunctional lipid repair.^199^ Class 3 ferroptosis inducers are distinguished by their action in depleting the GPX4 protein and endogenous lipophilic antioxidants, such as CoQ10, while simultaneously increasing the sensitivity of cells to ferroptosis. Caspase-independent lethal 56 (CIL56) and ferroptosis inducer 56 (FIN56) are two Class 3 ferroptosis inducers. The final class of ferroptosis inducers is composed of L-buthionine (S, R)-sulfoximine, which inhibits glutathione synthesis.^38^

**Table 1:** Pharmacological molecules interfering with ferroptosis

	Class	Mechanism	Pharmacological molecules
Inducer	Class 1	Inhibition of System Xc^−^	Erastin, sulfasalazine, sorafenib, artesunate, glutamate, lanperisone
	Class 2	Inhibition of GPX4	RSL3, Acetaminophen, withaferin A, FINO2
	Class 3	Depletion of GPX4 protein and CoQ10	FIN56, CIL56
	Class 4	Inhibitor of glutathione synthesis	L-buthionine (S, R)-sulfoximine^185^
Inhibitor	Class 1	Iron chelators/inhibitors of iron accumulation	Desferrioxamine (DFO), desferrioxamine mesylate, 2,2-bipyridyl,^184^ ciclopirox,^215^ di-2-pyridylketone 4,4-dimethyl-3-thiosemicarbazone (Dp44mT)^187^
	Class 2	Lipophilic antioxidants	Ferrostatin-1, ethyl 3-(benzylamino)-4-(cyclohexylamino)benzoate (SRS11–92),^185^ SRS 16–86,^188^ liproxstatin-1,^189^ Vitamin E,^190^ erythropoietin (EPO)^191^

Prohibiting ferroptosis has recently become a focus of treatment research, particularly for certain degenerative diseases. Regarding ferroptosis inhibitors, both lipophilic antioxidants and iron chelators suppress ferroptosis.^2^ Other compounds, such as D-PUFAs and lipoxygenase (LOX) inhibitors, have also been discovered. Ferrostatin-1, a ferroptosis inhibitor, has been shown to effectively improve the function of aged skeletal muscles, suggesting its potential for clinical translation as a therapeutic prospect.^200^ Although there is still a long way to go before inducers and inhibitors of ferroptosis can be used in clinical settings, recent research advances indicate the possibility for clinical intervention of degenerative orthopedic diseases.

### Targeting ferroptosis-sensitive factors in the diagnosis and treatment of degenerative orthopedic diseases

#### Reduction of the labile iron pool

When intracellular iron accumulates, excessive iron may initiate Fenton chain reactions to rapidly amplify the number of phospholipid hydroperoxides, which can then react with both Fe^2+^ and Fe^3+^ to produce additional free radicals, which further propagate phospholipid hydroperoxide production, ultimately triggering ferroptosis. Recent studies have revealed a close functional interplay between iron metabolism and ferroptosis.

Ferritin stores iron, and its concentration in serum or plasma reflects systemic iron storage, with a low ferritin level indicating iron deficiency and a high ferritin level reflecting the risk for iron overload. Therefore, serum and plasma ferritin concentrations are the most widely used indicators of systemic iron levels.^201^ For instance, serum ferritin levels have been used to diagnose iron-deficient heart failure with reduced ejection fraction.^202^ The WHO recommends serum or plasma ferritin concentrations as biomarkers for assessing iron status in individuals and populations.^203^ A common clinical complication in patients with β-thalassemia is osteoporosis, and serum ferritin is negatively correlated with BMD in β-thalassemia patients; therefore, serum ferritin level is an effective indicator of BMD in patients with severe and intermediate thalassemia.^204,205^ Excessive iron is also a hazard factor for lumbar disc herniation. Patients with severely degenerated discs have been shown to exhibit significantly higher serum ferritin levels than patients with mild symptoms, and serum ferritin levels were considerably greater in patients with Modic changes than in those without Modic changes.^177^ Osteoporosis is a comorbidity of menopause that is thought to be significantly influenced by estrogen deficiency. Clinical data have indicated that during the menopausal transition, the levels of estrogen decrease by 90%, while serum ferritin levels increase 2–3-fold.^206,207^ This finding informed a hypothesis suggesting that estrogen intervention may reduce the levels of ferritin, acting as an intervention factor of osteoporosis in postmenopausal women. In addition, alterations that lead to iron overload in joints increasing the risk of ferroptosis induction and osteoarthritic phenotype acquisition. Patients with high ferritin levels showed more severe joint damage,^208^ and imaging studies proved that the levels of serum ferritin was positively correlated with joint damage severity.^151,152^ Notably, a Cochrane meta-analysis revealed that blood ferritin concentration is a very sensitive indicator, with a detection threshold of 30 μg·L^−1^, and is a very specific marker of iron deficiency and that a high ferritin concentration is a sensitive indicator of iron overload; however, the reliability of these findings is low.^201^ Moreover, ferritin is an acute-phase protein, and ferritin levels are increased during inflammation and infection. The applicability of current WHO blood ferritin concentration thresholds to different age groups or physiological conditions is still limited. Hence, clinical and experimental validation is still needed to identify the appropriate blood ferritin concentration cutoffs. However, serum ferritin levels may be useful for a presumptive diagnosis and can guide efforts directed at further evaluation of osteoporosis and osteoarthritis.

The detrimental effects of excessive iron on the osteoarticular system and the development of degenerative bone diseases have been widely reported, although the pathogenic mechanisms underlying degenerative bone diseases in patients with iron overload have not been fully clarified,^121^ and ferroptosis is closely linked to the onset and progression of bone diseases. Therefore, reducing iron levels may help to clarify means of preventing or treating osteoporosis or osteoarthritis that is accompanied by iron overload. Furthermore, different treatments to reduce iron levels, such as iron chelators, may show therapeutic potential to prevent and/or overcome iron overload. Three iron chelators, DFO, deferasirox (DFX) and deferiprone (DFP), have been approved for clinical use.^209^ DFO was the first iron chelator approved for continuous intravenous or subcutaneous injection. DFX and DFP are oral iron chelators. All these iron chelators have been shown to effectively chelate iron,^210–212^ exhibit a good safety profile and promote compliance.^213,214^ DFO effectively reverses iron overload-induced chondrocyte apoptosis and extracellular matrix degradation in cartilage.^215^ Iron chelation therapy prevents osteopenia and/or osteoporosis in patients with thalassemia.^216,217^ Mohammadreza et al. compared the efficacy of five iron chelation therapies, namely, DFO, DFP, DFX, and their combination therapies, on bone loss in β-thalassemia patients.^218^ Their results showed that all five treatments attenuated bone loss in β-thalassemia patients, and the combination treatment consisting of DFX and DFO showed the greatest effect on reducing serum ferritin levels and improving BMD in the femoral neck and lumbar spine.^218^ Other clinical trials have confirmed these results, and this combination therapy decreased the levels of serum ferritin more efficiently and attenuated iron overload in the body.^219,220^ In addition, DFO prevented osteoporosis caused by other factors,^62,168^ with the underlying mechanisms related to the promotion of osteoblastic differentiation and the inhibition of osteoclast differentiation. For instance, iron overload causes osteoblast apoptosis and inhibits osteoblast differentiation, and DFO promotes the differentiation of osteoblasts via Wnt/β-catenin signaling.^221^ Osteoclasts require large amounts of iron to support mitochondrial biosynthesis and respiration during osteoclast differentiation, and therefore, iron chelation inhibits osteoclastogenesis and bone resorption.^168^

Calcium chelators also inhibit iron influx by modulating TFR1 internalization and are considered potential treatments for diseases caused by iron overload. BAPTA acetoxymethyl ester, a calcium chelator, inhibits iron influx into chondrocytes, thereby suppressing iron overload-induced ROS production and mitochondrial dysfunction.^222^ D-Mannose may as also be a ferroptosis inhibitor and exert chondroprotective effects by decreasing chondrocyte sensitivity to ferroptosis,^167^ thereby attenuating the progression of osteoarthritis.

Although these studies have shown that properly designed iron chelation interventions can prevent iron overload-related bone abnormalities, bone diseases remain an unresolved challenge and frequently cause complications in patients carrying excessive iron.^213,223^ Therefore, more research is needed to optimize chelation protocols in various iron overload situations and to explore alternative treatment strategies to reduce iron content. The aforementioned studies on iron chelators and calcium chelators will contribute to a better understanding of the therapeutic prospects of these compounds in ferroptosis-induced osteoporosis and osteoarthritis.

#### Suppression of lipid peroxidation

Lipid peroxidation is a key characteristic of ferroptosis that can lead to typical morphological changes, such as mitochondrial shrinkage. Phospholipids carrying polyunsaturated fatty acid (PUFA) chains are indispensable substrates that are susceptible to ferroptotic lipid peroxidation.^224^ Free PUFAs are likely to connect with membrane phospholipids, and ACSL4, which catalyzes the thioesterification of PUFAs along with coenzyme A to form their acyl-CoA derivatives, contributes to PUFA incorporation during in phospholipid metabolism, and therefore, decreased ACSL4 expression hinders the phospholipid-PUFA generation process and prevents ferroptosis.^225^ In ACSL4-deficient cells, the concentration of esterified PUFAs was far lower than that of free PUFAs.^37^ Many studies have focused on ACSL4 as a treatment of ferroptosis-related diseases. For instance, paeonol, a natural product isolated from Paeonia, significantly inhibited ferroptosis in heme-treated neuronal cells by inhibiting ACSL4 action,^226^ suggesting that paeonol may be a candidate drug for the treatment of degenerative orthopedic diseases. Rosiglitazone, a widely used peroxisome proliferator-activated receptor-γ (PPAR-γ) activator, inhibited lipid peroxidation and ferroptosis in smooth muscle cells and lung epithelial cells by inhibiting ACSL4 activity.^37,227,228^ PPAR-γ agonists inhibit MMP1 expression through a transcriptional mechanism and may help reduce joint tissue destruction.^229^ Moreover, low doses of rosiglitazone were shown to enhance the expression of type II collagen and TGF-β.^230^ Hence, rosiglitazone may exert a potential therapeutic effect on osteoarthritis.

LOX catalyzes the oxidative capacity of esterified PUFAs to generate lipid hydroperoxide LOOH, resulting in ferroptosis.^224^ LOX inhibitors, such as baicalein and zileuton, can increase cortical and total BMD, the trabecular area, and the number of trabecular nodes by inhibiting LOX activity and increasing the bone formation rate,^231^ making baicalein and zileuton promising therapeutic options for osteoporosis. Licofelone is an inhibitor of 5-LOX activity and is currently in clinical development for the treatment of osteoarthritis.^232^ It has been reported to modulate MMP13 production in human osteoarthritic chondrocytes,^233^ implying that licofelone may alleviate chondrocyte ferroptosis by inhibiting lipid peroxidation. Curcumin significantly reduces the expression of MMP3 and 5-LOX in the synovium, reduces serum malonaldehyde levels, and increases the levels of the antioxidant enzymes SOD, CAT and GPX, thereby attenuating osteoarthritis.^234^ Furthermore, a compound(s) in rose hip inhibits 5-LOX activity and proinflammatory metalloproteinases and shows the potential to be a treatment for osteoarthritis;^235^ that is, curcumin and rose hip may show the potential to alleviate chondrocyte ferroptosis by inhibiting LOX activity.

### Ferroptosis defense mechanisms in the diagnosis and treatment of degenerative orthopedic diseases

In the past 10 years, research on the defenses conferred by ferroptosis has been among the most rapidly developing areas of ferroptosis research. The cellular antioxidant system is the primary component of the defense system mediated by ferroptosis, and research into this defense system has important guiding significance for the clinical intervention of degenerative orthopedic diseases.

#### Activation of the System Xc^−^/GSH/GPX4 axis

System Xc^−^ is a heterodimeric amino acid transport complex that consists of two subunits, namely, the heavy chain 4F2hc protein (also known as SLC3A2) and the light chain xCT protein (also known as SLC7A11).^236,237^ SLC7A11 is a transporter protein with 12 putative transmembrane domains and is critical to the specificity of System Xc^−^; therefore, SLC7A11 is considered to be the core component of System Xc^−^. SLC3A2, which is a single-pass transmembrane regulatory protein, is crucial for maintaining the structural stability of System Xc^−^.^238^ System Xc^−^ exports intracellular glutamate in exchange for extracellular cystine in an ATP-dependent pattern,^239^ thereby rapidly reducing cystine to cysteine, which is required for GSH synthesis.^240^ The extracellular and intracellular concentrations of cystine and glutamate, respectively, maintain the normal function of System Xc^−^ to regulate the redox state of a cell, whereas an increased extracellular glutamate level can limit the intake of cystine and gradually lead to ferroptosis. Erastin, a ferroptosis inducer, is thought to inhibit system Xc^−^, resulting in decreased antioxidant capacity and an imbalance in intracellular lipid ROS contents.^241^ Hence, inhibiting System Xc^−^ leads to the induction of ferroptosis, and almost all regulators are directed against the core System Xc^−^ component SLC7A11. Recently, the overall structure of System Xc^−^ was analyzed via cryo-electron microscopy, and SLC3A2 was shown to interact with SLC7A11 mainly through hydrophobic and polar interactions at the extracellular interface and transmembrane region of cells; specifically, the Cys158 residue of SLC7A11 forms a disulfide linkage with the Cys211 residue of SLC3A2, and two lipid molecules bind to SLC7A11 near its intracellular transmembrane (TM) region.^242^ The residue immediately upstream of the transmembrane helix in SLC3A2 forms a short helix to anchor SLC7A11 to the intracellular side of cells.^242^ An area of nonprotein density in the intracellular vestibule of SLC7A11 was also observed at high resolution, and the shape of this area of density matched the shape of an erastin molecule, which was found to be sandwiched between the TM domains and integrated deeply into the intracellular vestibule of the SLC7A11 subunit, inhibiting cystine uptake and reducing intracellular GSH.^242^ The erastin analog imidazole ketone erastin docks in the same pocket as erastin and occupies a different pocket formed by residues Lys198, Ala247, Tyr251, and Ser330.^242^

LOOH, which can initiate peroxidative chain reactions, is an indispensable free radical precursor. Abundant radicals are derived from LOOH via slow molecule-assisted homolysis reactions or fast one-electron transfer reactions involving transition metals, such as Fe^2+^.^240,243^ The activity of GPX4 depends on the GSH level, and GPX4 inhibits ferroptosis by converting reduced GSH into oxidized glutathione disulfide (GSSG) and reducing lipid peroxides (LOOH) to lipid alcohols (LOH) to minimize free radical-related injury.^196^ GPX4 is required for the conversion of lipid peroxides into nontoxic fatty alcohols and for preventing excessive lipid hydroperoxide accumulation. The upregulation of GPX4 expression helps prevent iron-dependent toxic lipid ROS generation and accumulation during ferroptosis,^244^ while the inactivation of GPX4 accelerates lipid free radical overload and leads to ferroptosis. As a reduced substrate of GPX4, GSH is essential for inhibiting ferroptosis.^245^ In addition to promoting GSH synthesis, cystine uptake mediated by System Xc^−^ promotes the protein synthesis of GPX4. Cysteine activates rapamycin complex 1 (mTORC1) to promote GPX4 synthesis through the Rag-mTORC1–4EBP pathway, and pharmacological inhibition of mTORC1 activity decreases the level of GPX4 protein and enhances the sensitivity of cells to ferroptosis.^246^

Studies have demonstrated the potential effects of inhibitors or activators of System Xc^−^/GSH/GPX4 on the regulation of ferroptosis. Erasin, a ferroptosis inducer, induces ferroptosis in sensitive cells by reducing intracellular GSH levels. RSL3 is a ferroptosis inducer that inhibits GPX4 expression in a time- and dose-dependent manner and increases the cellular levels of unstable labile iron and ROS;^247^ moreover, ferroptosis can be induced by incompetent antioxidants and ROS aggregation. In osteoporotic rats, the GSH content and antioxidant enzyme activity were decreased; however, treatment with chrysin or naringenin increased the GSH level, improved bone quality, reduced bone resorption, and increased bone mineral deposition.^248^ Ginsenoside Rb2 and myricitrin reduce blood malondialdehyde (MDA) activity, increase GSH levels in mice, improve trabecular bone microstructure, and increase bone mineral density,^249,250^ indicating their great potential to inhibit ferroptosis, alleviate bone loss, and prevent osteoporosis. Melatonin exerts numerous physiological effects, including regulatory effects on circadian rhythms, immune defenses, and bone metabolism. Previous studies have suggested that melatonin can increase bone density and reduce the incidence of osteoporosis.^251^ Further mechanistic studies revealed that melatonin significantly inhibits ferroptosis via the Nrf2/SLC7A11/GPX4 pathway, thereby increasing the osteogenic ability of osteoblasts, and alleviates type 2 diabetes-induced osteoporosis.^252^ According to the literature available to date, the optimal concentration of melatonin for the treatment of osteoporosis remains unclear, and further clinical and experimental investigations are needed to verify the application prospects of melatonin.

Extracellular vesicles are important transporters that mediate intercellular communication and participate various physiological and pathological processes. Many studies have confirmed that stem cell-secreted extracellular vesicles play a protective role similar to that of stem cells and may be used in promising and effective therapy. Extracellular vesicles derived from induced pluripotent stem cells (iPSCs) and mesenchymal stem cells (MSCs) have been extensively studied in recent years.^253,254^ For instance, BM-EPC-derived extracellular vesicles have been shown to increase the expression of GPX4 and SLC7A11 and attenuate the pathological changes in the steroid-induced osteoporosis context by suppressing ferroptotic pathway activity.^144^ Therefore, cell therapy or therapy with cell-specific extracellular vesicle may be approaches to alleviate ferroptosis-related osteoporosis.

In osteoarthritis, icariin inhibits ferroptosis through the Nrf2/System Xc^−^/GPX4 axis and alleviates synoviocyte damage,^165^ suggesting that icariin can be exploited as a novel therapeutic candidate for synovitis. Quercetin increased the expression of GSH and GPX4 in rats with osteoarthritis by reducing the levels of MMP3 and MMP13 and maintaining the integrity of the extracellular matrix of articular cartilage.^255^ Nifedipine, a voltage-gated calcium channel inhibitor, activates the Nrf2 pathway to reduce oxidative stress, prevent cartilage degeneration and improve osteoarthritis.^256^ Exosomes derived from bone MSCs can regulate chondrocyte glutamine metabolism by regulating c-MYC and improving chondrocyte functional factors, such as type II collagen and aggrecan, thereby alleviating osteoarthritis. Although these studies did not directly address ferroptosis, considering the critical function of GSH/GPX4 in ferroptosis, it is reasonable to speculate that these intervention strategies are likely to be used in future treatments of osteoarthritis by inhibiting ferroptosis through activation of the GSH/GPX4 pathway.

#### Supplementation of the CoQ10/FSP1 system

Although activating GPX4 to inhibit ferroptosis has been recognized as a therapeutic strategy, many cells show resistance to GPX4 inhibitors, indicating that other factors may control their sensitivity to ferroptosis. FSP1, originally called apoptosis-inducing factor mitochondria-associated 2 (AIFM2), is a recently recognized antiferroptotic gene. Compared to normal cells, FSP1-knockout cells were more sensitive to ferroptosis inducers, such as the System Xc^−^ inhibitor erastin and GPX4 inhibitor ML162.^23^ FSP1 myristoylation facilitates its recruitment to lipid droplets and the cell membrane, where it functions as an NADH-dependent coenzyme Q10 (CoQ10) oxidoreductase and suppresses lipid peroxidation and ferroptosis via CoQ10 reduction. Even when GPX4 is functional, FSP1 deficiency leads to enhanced phospholipid oxidation, indicating that FSP1, which is a crucial element of the CoQ10 antioxidant system, functions in parallel with the classical GSH/GPX4 pathway. Pharmacological activation of FSP1 clearly cooperates with GPX4 to suppress lipid peroxidation and ferroptosis.^257^ Therefore, it has been proposed that inducers of the CoQ10/FSP1 system may exhibit potent anti-ferroptosis activity.

Ferroptosis-induced bone loss mediated by osteoblasts is associated with oxidative stress, indicating that dietary compounds with antioxidant properties are promising interventions. Previous studies have reported that short-term CoQ10 intake prevented the decline in bone mineral content and BMD caused by spinal cord injury,^258^ and long-term supplementation with CoQ10 and unsaturated fatty acids also increased BMD,^259^ suggesting that lifelong supplementation with CoQ10 and unsaturated fatty acids shows good potential for preventing osteoporosis and maintaining bone strength.

Previous reports have shown that CoQ10 exerts a profound anti-arthritic effect.^260,261^ Further mechanistic studies have shown that CoQ10 reduces RANKL-induced osteoclastogenesis and osteoclast differentiation and alleviates arthritis in mice.^262^ By decreasing inflammatory mediators and metalloproteinase activity, CoQ10 also reduces the degeneration of cartilage tissue in osteoarthritic joints and exerts a therapeutic effect on osteoarthritis cartilage degeneration.^263^ These advances imply that coenzyme Q10 may alleviate ferroptosis and may become a valuable therapeutic agent for osteoarthritis.

## Crosstalk between ferroptosis and other cell death modality pathway components

Although ferroptosis is different from other forms of cell death, the uniqueness of ferroptosis has been called into question in recent studies, which have argued that ferroptotic factors engage and factors involved in other cell death modalities, including autophagy and pyroptosis. This crosstalk may provide additional clues to advance pathogenesis research and develop clinical interventions in age-related degenerative bone diseases.

### Crosstalk between ferroptotic and apoptotic components

A series of important molecules in cells, such as proteins, lipids, and carbohydrates, are synthesized in the endoplasmic reticulum (ER). Therefore, ability to respond to ER function disruption is fundamental and critical to all living cells; however, ER stress can result in apoptosis. In multicellular eukaryotes, inositol-requiring protein 1 (IRE1), activating transcription factor 6 (ATF6), and protein kinase RNA (PKR)-like ER kinase (PERK) are the three most important upstream signaling proteins sensing ER stress, and when these three signaling molecules are activated, they induce ER stress-related apoptosis.^264^ Previous studies have shown that the ferroptotic agent erastin induced the blockade of glutamate-cystine exchange to activate the ER stress response and induce the expression of several proapoptotic proteins,^265^ indicating crosstalk between apoptosis and ferroptosis. In response to ER stress, erastin activates the PERK-eIF2α-ATF4-CHOP pathway, and the activation of C/EBP homologous protein (CHOP) results in the increased expression of p53 upregulated modulator of apoptosis (p53-independent PUMA), which participates in synergistic effects in ferroptosis and apoptosis.^266^ Although erastin induces the expression of the proapoptotic protein PUMA, it exerts no direct effect on cellular apoptosis, and the hallmark characteristic of apoptosis, poly (ADP-ribose) polymerase family member 1, is not activated during erastin treatment. Erastin-induced PUMA remained inactive in cells treated with erastin alone, but ferroptosis inducers activated PUMA when cells were cotreated with erastin and tumor necrosis factor-related apoptosis-inducing ligand (TRAIL).^266^ The activation of PUMA by ferroptotic agents may explain the molecular mechanism by which ferroptosis exacerbates apoptosis.

The lncRNA LINC00618 promotes apoptosis by increasing the expression of cleaved caspase-3 and BCL2-associated X (BAX) and induces ferroptosis by increasing the levels of iron and lipid ROS and decreasing SLC7A11 expression.^215^ Interestingly, LINC00618 accelerates ferroptosis in an apoptosis-dependent manner.^215^ These findings indicate that apoptosis and ferroptosis are mutually regulated. Advanced glycation end products (AGEs) have been shown to be pathogenic factors in osteoporosis in several studies, especially in diabetes-related osteoporosis. AGEs can induce both ferroptosis and apoptosis in human osteoblasts and disrupt the functions of osteoblasts. Furthermore, DFO inhibits AGE-induced ferroptosis and apoptosis.^267^ These studies indicate that the interaction of apoptosis and ferroptosis might be the key mechanisms in the pathophysiology of osteoporosis, and interventions targeting the key molecules common to ferroptosis and apoptosis will be beneficial for the clinical treatment of osteoporosis.

### Crosstalk between ferroptotic and autophagic components

Cytoplasmic misfolded or folded cytoplasmic proteins or organelles are engulfed by vesicles, which subsequently fuse with lysosomes to form autophagolysosomes in a process known as autophagy. Subsequently, the contents of the autophagolysosome are degraded or recycled to satisfy the metabolic needs and renew some organelles of cells. Autophagy is activated by both physiological and pathological processes in the body and is critical in many diseases, such as cancer and degenerative diseases. During age-related disc degeneration, for example, aberrant expression of Beclin-1 and LC3-II in NP cells was observed along with an abnormal number of autophagosomes. Similarly, degenerated human AF tissue showed markedly increased expression of autophagy-related genes, as well as increased amounts of autophagic vacuoles and autophagolysosomes, supporting the idea that dysregulation of autophagy pathways may be closely related to disc diseases.^268–270^

Many environmental stresses, such as nutrient deprivation, ER stress and oxidative stress, can trigger autophagy.^271^ Both of the ferroptosis inducers artesunate and erastin have been reported to induce the formation of autophagosomes mediated via the ER stress response.^272^ Autophagic degradation of ferritin produces labile iron, a process called ferritinophagy, which can promote ferroptosis.^273^ The results of RNAi screening and genetic analysis revealed that a number of autophagy-related genes positively regulated ferroptosis, and inducers of ferroptosis also activated autophagy and the subsequent autophagic degradation of ferritin.^274^ The inhibition of ferritinophagy and silencing of autophagy-related 7 (Atg7), Atg5, or nuclear receptor coactivator 4 (NCOA4) inhibited ferroptosis-associated labile iron and ROS accumulation and ultimately ameliorated ferroptosis.^274,275^ Notably, inhibition of NCOA4, a specific cargo transporter involved in the delivery of ferritin to lysosomes during ferritinophagy, inhibited the degradation of ferritin and prevented ferroptosis, whereas overexpressing NCOA4 facilitated ferritin degradation and accelerated ferroptosis.^273,275^

Yang et al. showed that ferritinophagy contributed to TBHP-induced autophagy and ferroptosis in NP and AF cells, and Fer-1 and DFO counteracted TBHP-induced ferritinophagy and ferroptosis.^176^ Silencing of NCOA4 alleviated ferroptosis by increasing the levels of GPX4 and FTH, whereas overexpression of NCOA4 aggravated ferroptosis, suggesting a possible role for NCOA4-mediated ferritinophagy and subsequent autophagic degradation of ferritin and lipid peroxidation during intervertebral disc degeneration.^176^ Elevated levels of NCOA4-related ferritinophagy and autophagy may, at least in part, maintain abnormal iron homeostasis during intervertebral disc tissue degeneration. Further investigation of the crosstalk between ferroptosis and autophagy in age-related degenerative orthopedic diseases is warranted to deeply explore the pathogenesis and develop effective ferroptosis-specific inhibitors for clinical treatment and prophylaxis.

### Crosstalk between ferroptotic and pyroptotic components

Pyroptosis is another form of programmed cell death that is frequently associated with inflammation. Pyroptosis is characterized by cell lysis, swelling, and release of proinflammatory cytokines, including IL-18 and IL-1β. Caspases that are activated by cleavage, such as cleaved caspase-1, can further cleave the hinge region between the C- and N-terminal domains of gasdermin E (GSDME) or GSDMD, and then GSDMD-N is inserted into the lipid bilayer of cell membranes through its N-terminal domain to form pores that induce osmotic cell lysis, inducing pyroptosis.^276,277^ Pyroptosis is activated through two main pathways: the caspase-3/GSDME pathway and the GSDMD-dependent pathway regulated by caspase 1/4/5/11.^276–280^ Pyroptosis plays an important role in various diseases, particularly degenerative bone diseases.^21,281^ Increased inflammatory activity caused by pyroptosis in joints promotes cartilage degeneration and exacerbates the progression of synovitis and osteoarthritis,^21^ which are associated with the release of inflammatory factors, leading to excessive osteoclast differentiation and excessive bone resorption.^282^ For example, the levels of cleaved caspase-3 and GSDME-N in monocytes and synovial macrophages were increased in patients with rheumatoid arthritis and were positively correlated with disease activity; moreover, blocking pyroptosis effectively alleviated arthritis in mice.^283^

Although ferroptosis and pyroptosis are distinguished by their unique characteristics, recent advances in cancer therapy have suggested that components in these pathways may engage in crosstalk.^284^ For example, antitumor immune cells, represented by CD8^+^ T cells, simultaneously promote and induce these two types of cell death. On the one hand, CD8^+^ T cells promote the activation of GSDMB and induce pyroptosis;^285^ on the other hand, IFN-γ secreted by CD8^+^ T cells downregulates SLC3A2 and SLC7A11, causes lipid ROS accumulation, and induces ferroptosis.^286,287^ Ultraviolet B (UVB) radiation triggered both ferroptosis and pyroptosis in epidermal keratinocytes; furthermore, the ferroptosis inhibitor Fer-1 inhibited caspase 1 activation and its cleavage of pro-IL-1β and GSDMD; hence, it alleviated UVB-induced skin inflammation.^288^ In septic lung injury mice, both pyroptosis and ferroptosis were found to be involved in acute lung injury.^289^ In addition to reducing the level of pyroptosis-related proteins, such as GSDMD-N, the expression of GPX4 was increased, and the expression of PTGS2 and 4-HNE was decreased, indicating that the inhibition of pyroptosis suppressed ferroptosis. Moreover, in addition to increased GPX4 and decreased PTGS2 levels, the expression levels of pyroptosis-related proteins was also decreased after Fer-1 treatment, suggesting that blocking ferroptosis also inhibited pyroptosis.^289^ These findings demonstrate a potential interaction between ferroptosis and pyroptosis pathways, but the exact mechanism remains unclear. Further investigations are necessary to probe new evidence of extensive molecular crosstalk between cell death pathways in age-related bone diseases, describe the physiological and pathological processes of these interactions, and fully understand the potential clinical implications.

## Conclusion and perspective

Recently, ferroptosis, as a novel type of cell death, has become an important topic of discussion. Ferroptosis plays a crucial role in regulating bone homeostasis and regeneration and exhibits desirable curative prospects. Iron overload and oxidative stress are associated with cartilage degradation or abnormal bone metabolism, which may indicate an opportunity for targeting ferroptosis to treat age-related orthopedic diseases.

However, several questions remain unanswered by recent research. Studies and clinical trials on the molecular biology-related mechanisms of ferroptosis have only scratched the surface of this programmed death process, and because of this dearth of molecular information, it is relatively difficult to identify ferroptosis at an early stage. Even the most prominent morphological damage to the mitochondrial structure is a challenge to detect precisely because biomarkers exclusively associated with ferroptosis have not been identified to date.^290^ Although mitochondria are clearly intimately involved in ferroptosis, an overall understanding of the mitochondrial molecular mechanisms is debated.^291^ Although ferroptosis participates in most pathological stages of degenerative orthopedic diseases, and it is accompanied by the concentration of specific mutable genes or proteins that influence the development of ferroptosis, such as GPX4, the testing technology available is unsuitable and fails to meet routine clinical diagnostic needs.^292^ Therefore, no suitable biomarkers are available for the prevention or prognostication of age-related orthopedic diseases. Although ferroptosis exhibits unique features that distinguish it from other cell death modalities, such as autophagy, necrosis, and apoptosis, the ferroptosis inducer erastin also induces caspase-9-dependent mitochondrial apoptosis, and activation of the autophagy signaling pathway has also been observed in ferroptotic cells, suggesting crosstalk among these cellular phenotype-related pathways. Moreover, core regulators of ferroptosis, such as SLC7A11, GPX4, Nrf2, and P53, have been implicated in apoptosis or autophagy. Therefore, understanding the network organization of the ferroptosis system, rather than the effects of a single regulators, is more important for an in-depth understanding of the mechanisms underlying ferroptosis. Findings clarifying the ferroptotic network will also provide clues for the treatment and diagnosis of age-related orthopedic diseases.

Further research is warranted to clarify the latent mechanisms underlying ferroptosis in the occurrence and development of age-related orthopedic diseases. We hope that more effective and appropriate strategies for clinical treatment and prophylaxis will emerge in the near future.
